# The involvement of spinal annexin A10/NF-κB/MMP-9 pathway in the development of neuropathic pain in rats

**DOI:** 10.1186/s12868-019-0513-9

**Published:** 2019-06-17

**Authors:** LiHong Sun, Qi Xu, WenXin Zhang, CuiCui Jiao, Hui Wu, XinZhong Chen

**Affiliations:** Department of Anesthesiology, Women’s Hospital, School of Medicine, Zhejiang University, Hangzhou, 310000 Zhejiang Province China

**Keywords:** Pain, ANXA10, NF-κB, MMP-9, Spinal cord, Cytokines

## Abstract

**Background:**

Neuropathic pain (NP) is a prevalent disease, which badly impairs the life quality of patients. The underlying mechanism of NP is still not fully understood. It has been reported that spinal Annexin A10 (ANXA10) contributes to NP. This study aims at exploring the underlying mechanisms of spinal ANXA10 in regulating NP in rats.

**Methods:**

Spinal nerve ligation (SNL) was adopted to establish a NP model in rats. After SNL, paw withdrawal threshold and paw withdrawal latency were recorded to measure pain behaviors, RT-PCR was used to check the change of the expression of spinal ANXA10 mRNA, western blot analysis was used to detect the change of the protein level of ANXA10, nuclear factor kappa B (NF-κB), and maisrix metalloproteinase-9 (MMP-9) in the spinal cord. The levels of proinflammatory cytokines, including tumor necrosis factor-α (TNF-α), interleukine-1β (IL-1β), and interleukine-6 (IL-6), were explored by ELISA kits. The effects of both knockdown of spinal ANXA10 and inhibition of NF-κB on pain behaviors and the expression of MMP-9 and proinflammatory cytokines were investigated.

**Results:**

Our present findings highlighted that SNL caused pain hypersensitivity and increased the expression of spinal ANXA10/pNF-κB, TNF-α, IL-1β, and IL-6 both in the early and late phase of NP in rats, while spinal MMP-9 was only slightly increased in the early phase of NP. Knockdown of ANXA10 at the spinal cord level suppressed the SNL-induced hyperalgesia and blocked the activation of NF-κB, TNF-α and IL-1β both in the early and late phase of NP. Spinal ANXA10 knockdown could prevent the upregulation of spinal MMP-9 in the early phase and inhibit IL-6 expression in the late phase of SNL-induced NP.

**Conclusions:**

In conclusion, spinal ANXA10/NF-κB/MMP-9 pathway, along with the activation of proinflammatory cytokines, was involved in the SNL-induced NP. MMP-9 may act as the downstream target of ANXA10/NF-κB pathway in the development rather than the maintenance of NP.

## Background

Neuropathic pain is a common pain state, caused by primary lesion or disease in the central or peripheral nervous system. Characterized by hyperalgesia (enhanced pain response to a normally painful stimulus) and allodynia (painful response to a normally harmless stimulus), NP is an intractable disease worldwide [1]. The underlying pathophysiological mechanisms of neuropathic pain are still not fully understood. The spinal cord, which receives input from peripheral nociceptors and projects to the upper central nervous systems, plays a pivotal role in the progress of pain [2]. It is generally believed that the alteration of spinal gene expression and pain-related signal pathway is an important factor in the formation and development of chronic neuropathic pain [3]. Annexin A10 (ANXA10) is the latest identifed member of annexin family, which is an evolutionary conserved multigene family formed by calcium and phospholipid binding proteins [4]. Former studies have shown that ANXA10 is closely related to some physiological and pathological processes including tumor progression, cell division, and dedifferentiation. ANXA10 may act as a possible tumor suppressor role in gastroenteric cancers [5]. Recently, Lu et al. found that in a mice model of SNL-induced neuropathic pain, spinal ANXA10 gene is highly upregulated in the astrocytes and neurons and is involved in the pathogenesis of neuropathic pain [6]. It has been reported that spinal extracellular regulated kinase (ERK) signalling and the subsequent release of proinflammatory cytokines act as the downstream pathway of ANXA10 in modulation of neuropathic pain caused by chronic constrictive injury in rats [7].

Pain-related cytokines participate in pain modulation at the spinal cord level through autocrine and paracrine, leading to hyperalgesia. Activated glial cells are the main source of cytokines in the central nervous system (CNS). When glial cells are activated, they produce and release a large number of cytokines such as IL-1β, IL-6, and TNF-α [8, 9]. Some proinflammatory cytokine are well known to be key modulators in the pathogenesis of neuropathic pain. The signaling pathways related to cytokines include the nuclear factor kappaB (NF-κB) transduction pathway. NF-κB is a homologous or heterodimeric complex, among which p50/p65 is the most common dimeric form. NF-κB is an important transcription factor that regulates the gene expression of multiple pain-related factors [10]. Constitutively expressed in CNS, NF-κB plays a vital role in cell survival and synaptic plasticity. Activation of NF-κB is triggered in many kinds of animal models of neuropathic pain such as spinal nerve ligation, chronic constriction injury of the sciatic nerve [11]. Pain sensitivity caused by nerve injury can be alleviated by inhibition of NF-κB. Translocation of NF-kB into the nucleus could trigger the activation of many cytokines, including IL-1β, IL-6, TNF-α [12].

MMP-9 is a member of maisrix metalloproteinase (MMP), which is a group of Zn^2+^-containing proteases that degrade the extracellular matrix (ECM) and participate in the inflammation and tissue repair processes of various neurodegenerative diseases [13, 14]. Many studies have demonstrated that expression of MMP-9 in dorsal root ganglion (DRG) and spinal cord was correlated with the pathological process of neuropathic pain [13, 15]. The mechanisms of MMP-9 in mediating neuropathic pain are complex. It’s been reported that the binding sites of MMP-9 on its promotor was regulated by NF-κB [16]. The NF-κB/MMP-9 signaling pathway has been implicated in many biological processes, such as injury-induced neointimal hyperplasia, the process of cancer cell invasion [17]. There is increasing evidence to support the idea that NF-κB/MMP-9 pathway is also involved in the development of neuropathic pain [18]. Besides, MMP-9 has been shown to induce neuropathic pain through promoting cleavage of some particular cytokines at early times after nerve injury [19].

In this study, we performed spinal nerve ligation (SNL) model, which is commonly believed to mimic the human patients suffering from long-lasting and recurrent pain. We focused on NF-κB/MMP-9 pathway and the release of proinflammatory cytokines to dissect out the underlying mechanism of ANA10 in modulating neuropathic pain.

## Methods

### Animals

Adult male Sprague–Dawley rats weighing 200–250 g were used in the current study. The rats were purchased from Zhejiang Chinese Medical University (Hang Zhou, China). All the animals were maintained under a 12 h/12 h light/dark cycle with a constant room temperature (RT) of 23 ± 1 °C. All experiments were performed according to the guidelines and regulations set by the International Association for the Study of Pain.

### Spinal nerve ligation (SNL)

Firstly, the rats were anesthetized with sodium pentobarbital (40 mg/kg) intraperitoneally. After the skin was sterilized, a midline incision was made along the sixth left lumbar. Then the left paraspinal muscles were separated and L_6_ transverse process was removed to expose the left L_5_ spinal nerve. The L_5_ spinal nerve was carefully separated and ligated with 4–0 silk sutures. Finally, the incision was closed layer by layer. Rats in the sham group underwent the same procedure except for nerve ligation.

### Behavior tests

The mechanical thresholds were measured by paw withdrawal threshold (PWT). PWT was assayed by von Frey test as described by Chaplan et al. [20]. Before testing, rats were placed in a transparent plastic cage on wire mesh to adapt to the environment for 30 min. The procedure was performed while keeping surrounding environment quiet. Using Von Frey filament, we pressed the plantar surface of each rat’s left hind paw. When the rat showed paw withdrawal or paw-licking responses, a lower filament was switched. If there were no positive responses, a higher filament was selected. The withdrawal threshold was evaluated using Dixon’s up–down method.

Paw withdrawal latency (PWL) was used to determine the thermal hyperalgesia of rats using heat radiation method. Each rat was placed in a plexiglass box, which was placed on a glass plate, to adapt to the environment for 30 min. The plantar surface of left foot was irradiated with a heat stimulator in accordance with the Hargreaves method [21]. The time from the beginning of the irradiation to the emergence of paw withdrawal or paw-licking response was recorded as PWL. A cutoff time of 30 s was set to avoid skin or tissue damage.

### Quantitative reverse transcription polymerase chain reaction (RT-PCR)

The rats were anesthetized with sodium pentobarbital (50 mg/kg) intraperitoneally. The L_4–5_ region of the spinal cord was quickly dissected into separate RNase-free Eppendorf tubes and immediately transferred to − 80 °C refrigerator. Total RNA of the frozen lumbar spinal cord was isolated by using Trizol reagent (Life Technologies, CA, USA). After purification, the RNA concentration was estimated using the Spectrophotometer (Thermo Fisher Scientific). Real-time PCR was performed using the SYBR PrimeScript Kit (Takara) on the CFX96 Real-time PCR detection system (Bio-Rad, US). The primer sequences of rat ANXA10 were: Forward: TGATGGATGCCCAAGTGATAG, Reversd: CATTGCTGCGTTGTGTTAGG. β-actin was taken as reference gene. The primer sequences of β-actin were: Forward: AGGTCGGTGTGAACGGATTTG, Reversd: GGGGTCGTTGATGGCAACA. Relative RNA expression (fold change) was analyzed using the 2^−ΔΔCt^ method [22].

### Western blotting

The rats were anesthetized with sodium pentobarbital (50 mg/kg) intraperitoneally. The L_4–5_ spinal cord was rapidly removed and frozen in liquid nitrogen. The tissues were homogenated with protein lysate buffer for 30 min on ice. After the homogenate was centrifuged at 10,000 rpm for 15 min at 4 °C, the supernatants of the samples were collected and estimated for protein concentration by the BCA protein assay (Boster, Wuhan). After being boiled at 100 °C for 5 min, the supernatant was mixed with 5 × loading buffer. Samples containing equal amount of total protein were loaded and electrophoresed on a 10% SDS-PAGE gel. The separated proteins were then transferred to the polyvinylidene fluoride (PVDF) membranes. The membranes were blocked in 5% non-fat milk at room temperature for 2 h. Then, the membranes were incubated with primary antibody rabbit anti-ANXA10 antibody (1:1000, db2943, Diagbio, lot: Q1009); rabbit anti-pNF-κB/p65 (1:1000, ab86299, Abcam, lot: GR3204852-6); rabbit-anti-MMP-9 (1:1000, ab38898, Abcam, lot: 573144); rabbit-anti-GAPDH (1:2000, db106, Diagbio, lot: P1020) overnight at 4 °C. The membranes were washed for 3 × 10 min with TBST (Tris-buffered saline Tween-20). After being washed, the membranes were further incubated with secondary antibody (goat anti-rabbit IgG, HRP, 1:5000, BA1054, Boster) at room temperature for 2 h. Membranes were washed for 3 × 10 min with TBST. Quantity One (Bio-Rad,USA) was used to perform the densitometry analysis.

### Enzyme-linked immunosorbent assay (ELISA)

The levels of proinflammatory cytokines in the L_4–5_ spinal dorsal horn tissues were detected by the ELISA kits (TNF-α: ab46070, Abcam; IL-1β: ab100768, Abcam; IL-6: ab100772, Abcam) according to the attached instructions of the kits. Samples were assayed in triplicate so that each experiment was performed three times to take the average value.

### Administration of drugs

The drugs were administrated intrathecally in rats under inhalational anesthesia. Rats were briefly anesthetized with 2% sevoflurane, the lower back of the rat was shaved and sterilized. Using a Hamilton microsyringe, the drug was injected between the L_4_ and L_5_ vertebrae. A sudden slight flick of the tail implicated that the needle has just entered into the subarachnoid space. The drug was injected slowly in 30 s. After injecton, the needle was held in place for a further 10 s to prevent outflow. Finally, the incision site was closed in layers.

A small interfering RNA (siRNA) was designed to target rat ANXA10 based on the genomic sequence (Gene Bank Accession: NM_001109110.1). The targeted nucleotide sequences were: 5′-TGCGAGAAGCCTACTGTTTACAATACAGC-3′. A scrambled sequence was designed as negative control (NC siRNA). The siRNA (3 μg/μl, dissolved in 10 μl PBS) was injected intrathecally. PDTC (ab141406, Abcam, 0.5 μg/10 μl), was dissolved in dimethyl sulfoxide (DMSO) for intrathecal injection.

### Statistical analysis

All data were presented as mean ± SD. Statistical analyses were performed using GraphPad Prism 6 (USA). Student’s *t* test was used to compare the data between two groups of samples. One-way repeated analysis of variance (ANOVA) or two-way repeated ANOVA, followed by Bonferroni post hoc test was used to determine significance between different multiple groups. The difference was considered statistically significant if *p* < 0.05.

## Results

### Effects of SNL on behavioral tests and the expression of spinal ANXA10/NF-κB/MMP-9 and proinflammatory cytokines

**Fig. 1:**
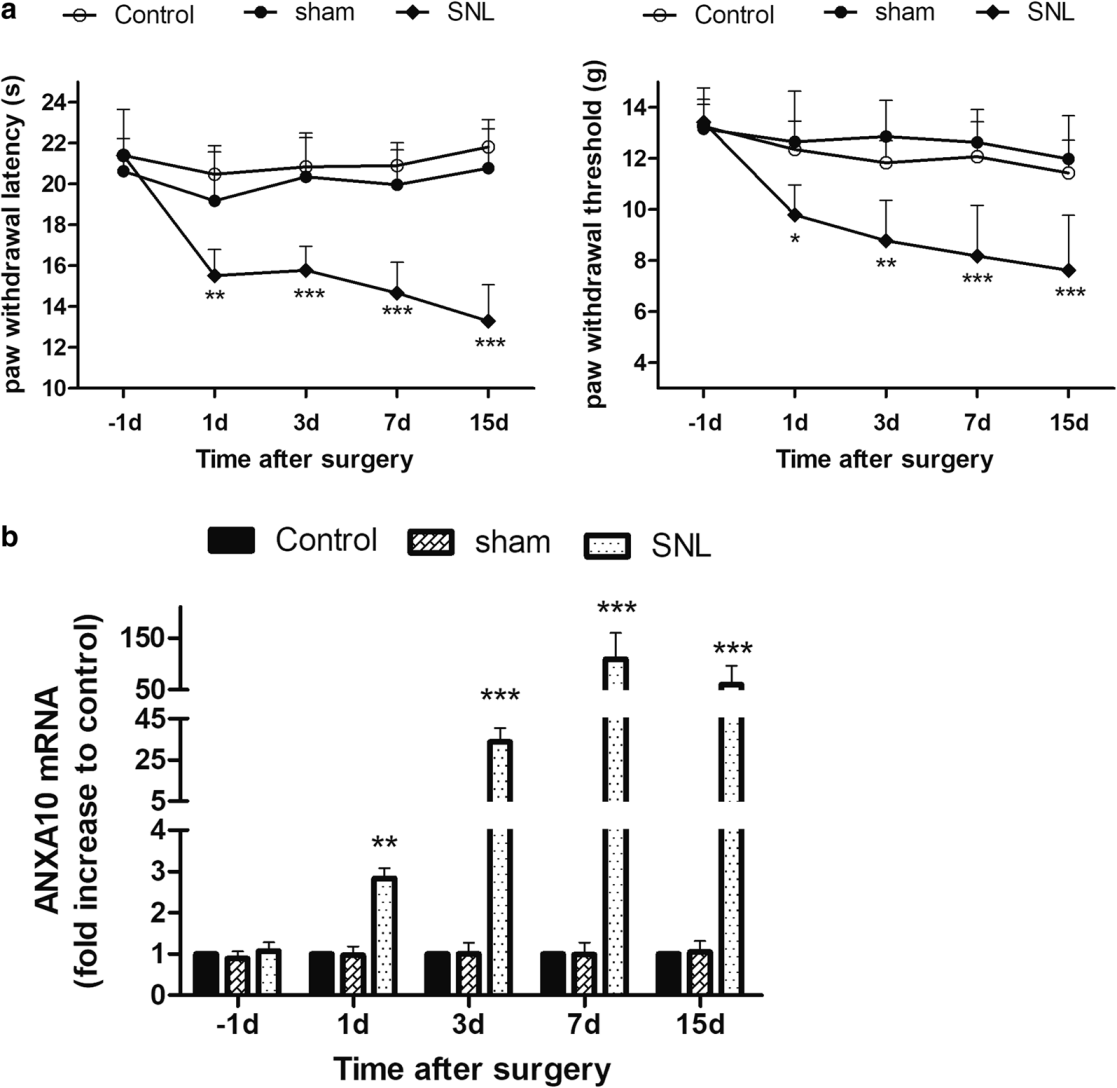
Behavioral tests and detection of the expression of spinal ANXA10 mRNA after SNL. **a** PWL and PWT measured on day 1 before surgery, day 1, 3, 7, and 15 after surgery in control, sham, and SNL group. (*p < 0.05, **p < 0.01, ***p < 0.001, compared with sham group, n = 6) **b** PCR analysis of the expression of ANXA10 mRNA in the spinal cord after surgery in control, sham, and SNL group. The L_4–5_ spinal cord tissue was extracted on day 1 before surgery, day 1, 3, 7, and 15 after surgery. (**p < 0.01, ***p < 0.001, compared with sham group, n = 6)

Behavioral testing (PWL and PWT) was undertaken on day 1, 3, 7, and 15 after SNL or sham surgery operation. The naive rats were used as control group. In sham group, the L_5_ spinal nerve of each rat was separated but not ligated. The results showed that SNL produced thermal and mechanical hyperalgesia, which developed on day 1 after surgery and sustained for more than 2 weeks (Fig. 1a). The change of spinal ANXA10 mRNA was checked by RT-PCR on day 1, 3, 7, and 15 after surgery. Compared to the sham group, SNL caused an upregulation of ANXA10 mRNA in the spinal cord (Fig. 1b). Both the results of behavior and PCR indicated that there was no significant difference between rats in control group and sham group, thus we included only sham group in the following experiments.

**Fig. 2:**
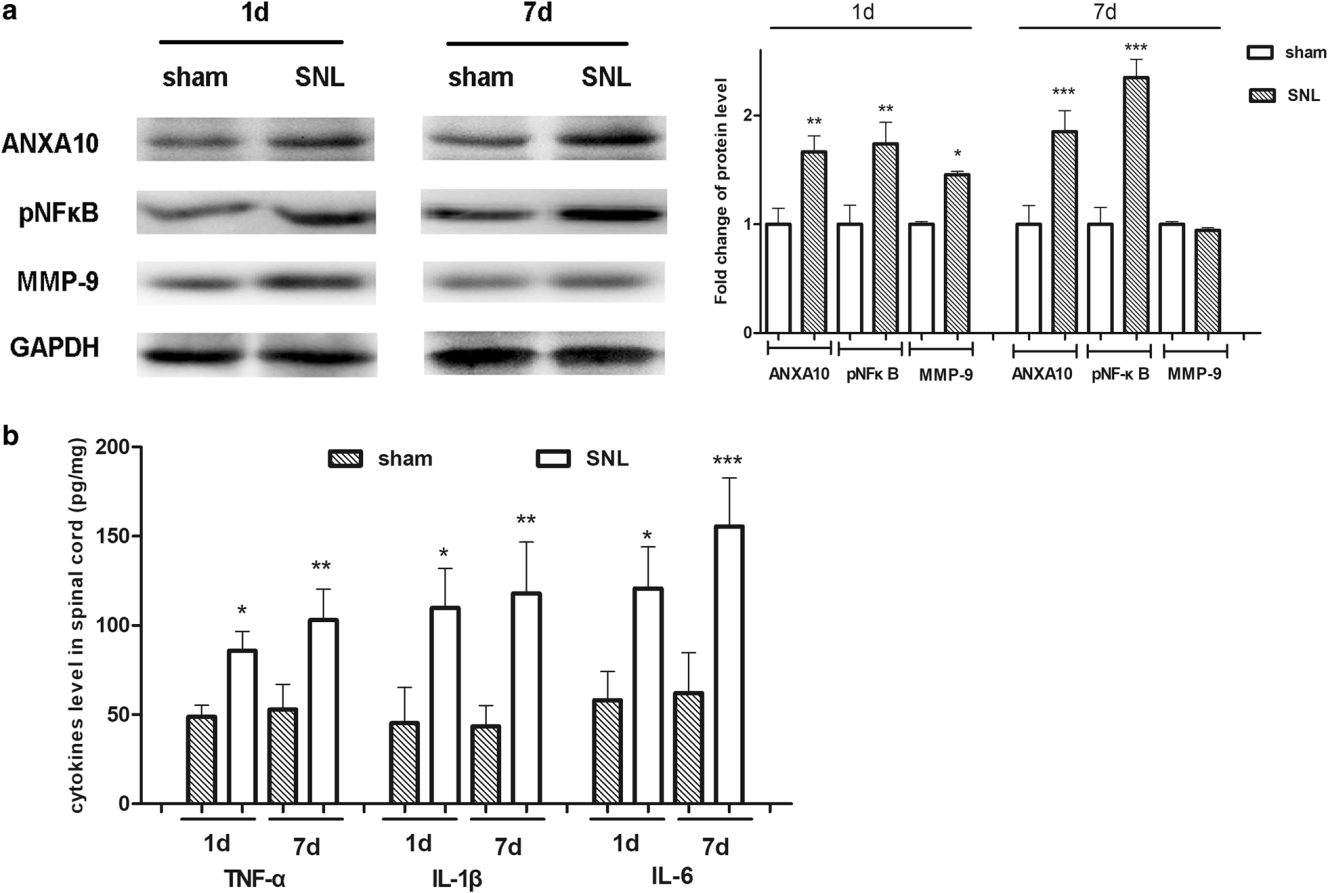
Detection of the expression of spinal ANXA10, pNF-κB, MMP9 and proinflammatory cytokines after SNL. **a** Western blot analysis of the expression of spinal ANXA10, pNF-κB, MMP9 on day 1 and day 7 after surgery in sham and SNL group. Fold change for the density of protein level was normalized to GAPDH. (*p < 0.05, **p < 0.01, ***p < 0.001, compared with sham group, n = 4) **b** ELISA analysis of the expression of spinal TNF-α, IL-1β, and IL-6 in sham and SNL group. (*p < 0.05, **p < 0.01, ***p < 0.001, compared with sham group, n = 4)

Western blot analysis showed that the expression of ANXA10 and pNF-κB (the activated form of NF-κB) in the L_4–5_ dorsal horn increased on day 1 and day 7 after SNL (Fig. 2a). There was a mild increase in the expression of MMP-9 on day 1 after SNL. However, on day 7 after surgery, there was no significant change of MMP-9 expression between sham group and SNL group (Fig. 2a). To search for cytokines involved in the process of neuropathic pain, we examined the expression of TNF-α, IL-1β, and IL-6 in the spinal dorsal horn. ELISA analysis showed that the expression of these cytokines increased both on day 1 and day 7 after SNL, which was in consistent with the results of former studies (Fig. 2b).

### Effects of siRNA ANXA10 on behavioral tests and the expression of spinal NF-κB/MMP-9 and proinflammatory cytokines

**Fig. 3:**
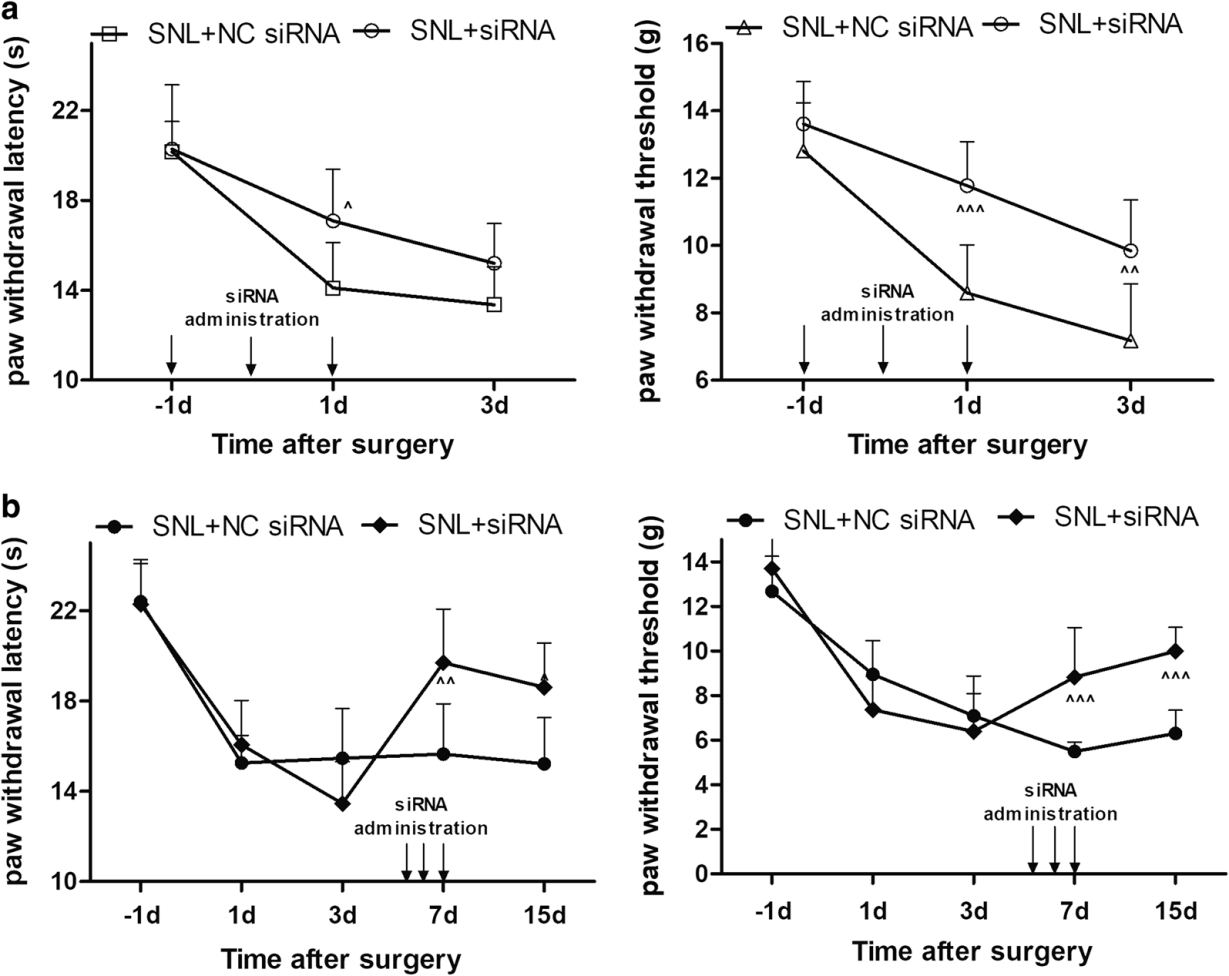
Behavioral tests after intrathecal administration of siRNA ANXA10. **a** PWL and PWT measured on day 1 before surgery, day 1, and 3 after surgery in SNL + NC siRNA, and SNL + siRNA group. The siRNA or NC siRNA was administrated intrathecally on day 1 before surgery, at 4 h before surgery, on day 1 after surgery. (^*p *< 0.05, ^^*p *< 0.01, ^^^*p *< 0.001, compared with “SNL + NC siRNA” group, n = 6) **b** PWL and PWT measured on day 1 before surgery, day 1, 3, 7, and 15 after surgery in SNL + NC siRNA, and SNL + siRNA group. The siRNA or NC siRNA was administrated on day 5, 6, 7 after surgery. (^^*p *< 0.01, ^^^*p *< 0.001, compared with “SNL + NC siRNA” group, n = 6)

In order to further investigate the role of ANXA10 in the modulation of neuropathic pain, the siRNA targeting ANXA10 was administrated intrathecally to knockdown spinal ANXA10 both in the early and late phase of SNL-induced neuropathic pain. Behavior results showed that knockdown of ANXA10 in the early phase (siRNA administrated continuously on 1 day before surgery, at 4 h before surgery, and on 1 day after surgery) alleviated the SNL-induced thermal hyperalgesia on day 1 after surgery, and inhibited the mechanical hyperalgesia for more than three days (Fig. 3a). Meawhile, inhibition of ANXA10 in the late phase (siRNA administrated consecutively on day 5, day 6, and day 7 after surgery) relieved both the thermal and mechanical hyperalgesia for more than 8 days (Fig. 3b).

**Fig. 4:**
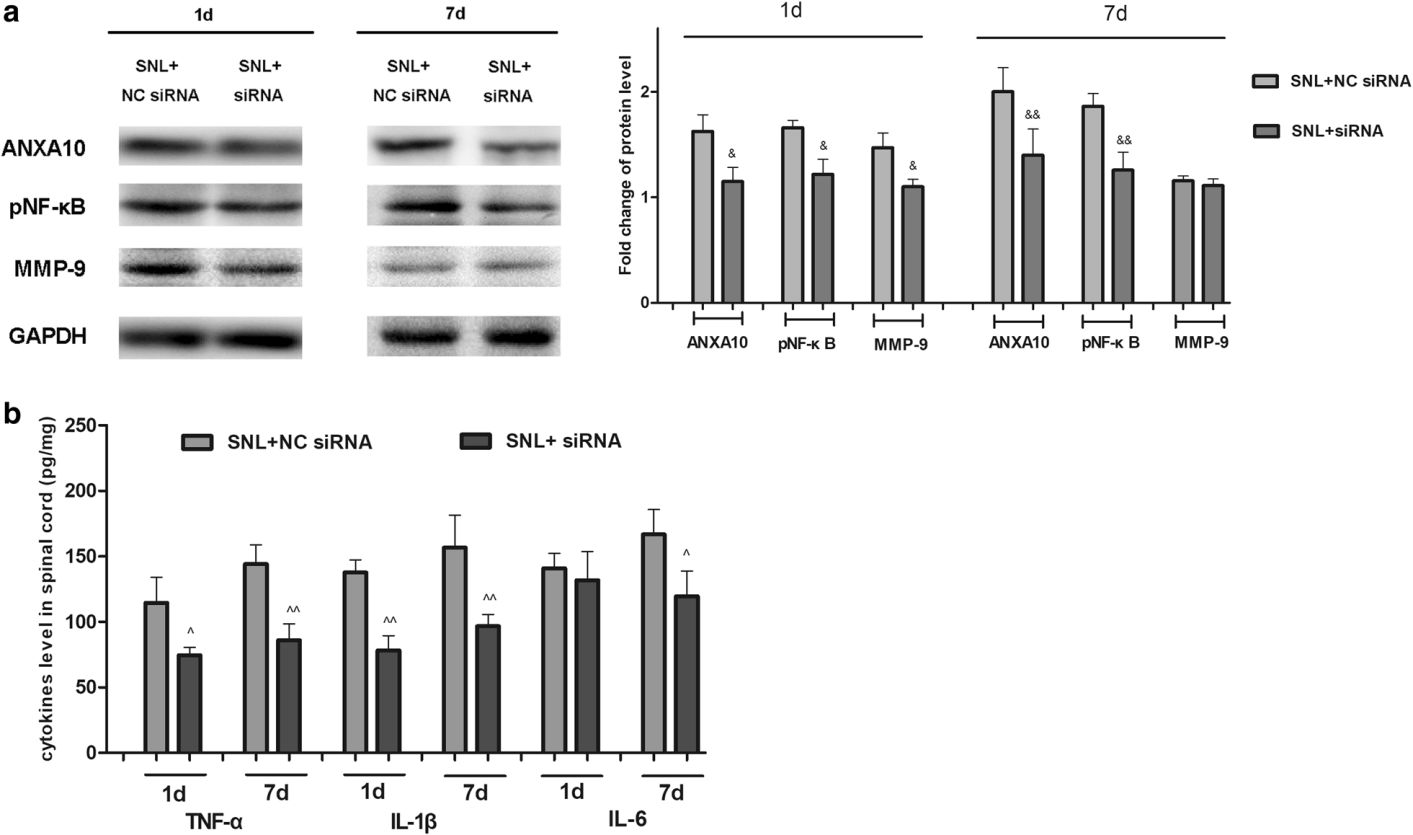
Detection of the expression of spinal pNF-κB, MMP9 and proinflammatory cytokines after intrathecal administration of siRNA ANXA10. **a** Western blot analysis of the expression of spinal ANXA10, pNF-κB, MMP9 on day 1 and day 7 after surgery in SNL + NC siRNA, and SNL + siRNA group. Fold change for the density of protein level was normalized to GAPDH. (^&^*p *< 0.05, ^&&^*p *< 0.01, compared with “SNL + NC siRNA” group, n = 4) **b** ELISA analysis of the expression of spinal TNF-α, IL-1β, and IL-6 in SNL + NC siRNA, and SNL + siRNA group. (^*p *< 0.05, ^^*p *< 0.01, compared with “SNL + NC siRNA” group, n = 4)

Western blot analysis showed that compared to NC siRNA treatment, siRNA administration in the early phase not only blocked the expression of ANXA10, but also prevented the expression of pNF-κB and MMP-9. Inhibition of ANXA10 in the late phase suppressed the activation of NF-κB, but had no effect on MMP-9 expression (7d after sugery) (Fig. 4a). The SNL-induced upregulation of spinal TNF-α and IL-1β was prevented by both early and late treatment of ANXA10 inhibition. Interestingly, the increased release of spinal IL-6 caused by SNL was suppressed by ANXA10 siRNA treatment in the late phase rather than early phase (Fig. 4b). These results indicated that in the early development of neuropathic pain, ANXA10 acted as an upstream regulator of the initial activation of NF-κB/MMP-9, and the subsequent release of TNF-α and IL-1β. In the late phase of neuropathic pain, ANXA10 promoted the activation of NF-κB, and the release of TNF-α, IL-1β, and IL-6. MMP-9 may be the downstream target of ANXA10 only in the early phase of neuropathic pain.

### Effects of NFκB inhibitor on the expression of spinal MMP-9 and proinflammatory cytokines

**Fig. 5:**
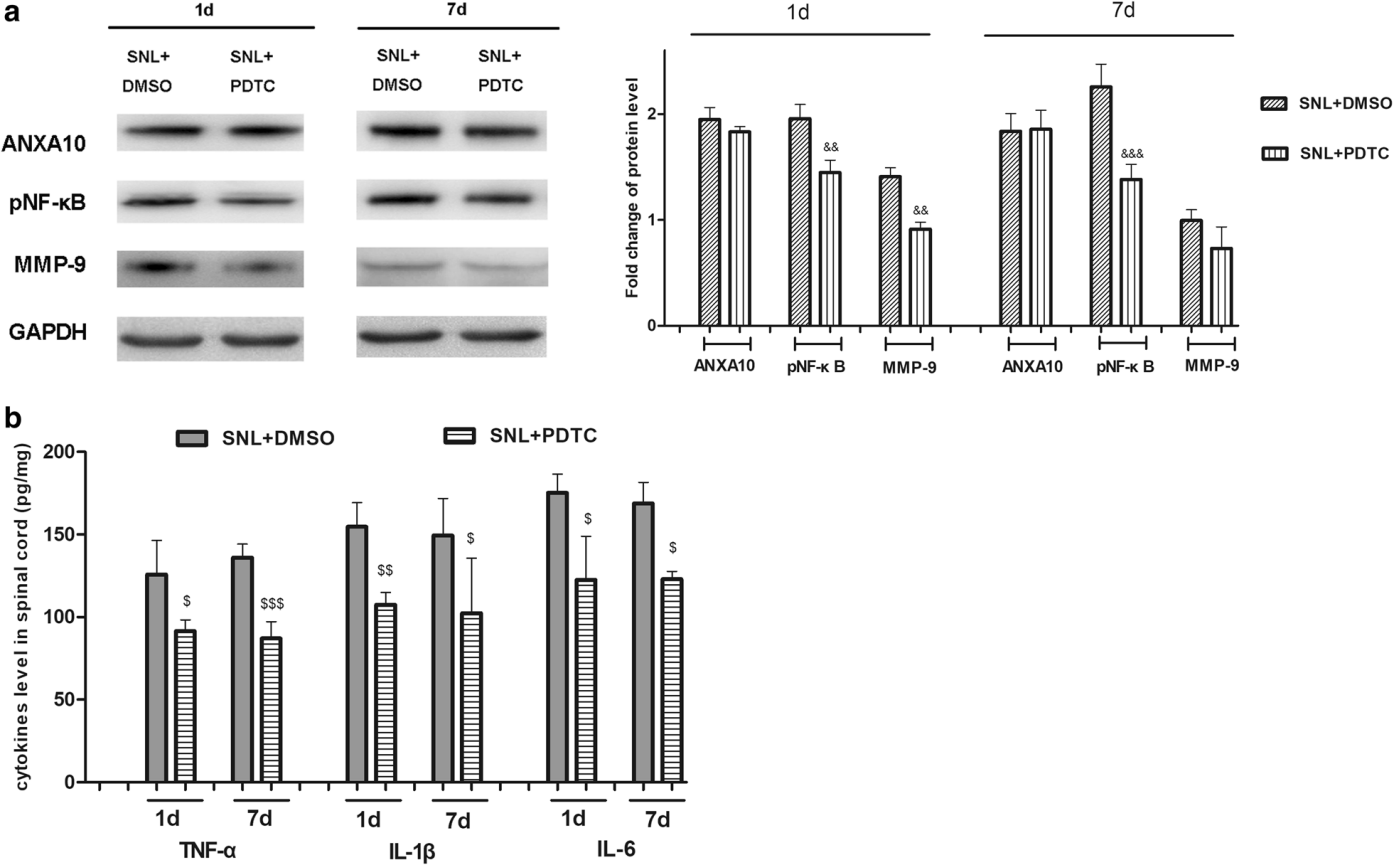
Effects of NFκB inhibitor (PDTC) on the expression of spinal MMP9 and proinflammatory cytokines. **a** Western blot analysis of the expression of spinal ANXA10, pNF-κB, MMP9 on day 1 and day 7 after surgery in SNL + DMSO, and SNL + PDTC group. The DMSO or PDTC was administrated on day 1 before surgery, at 2 h before surgery, on day 1 after surgery. Fold change for the density of protein level was normalized to GAPDH. (^&&^*p *< 0.01, ^&&&^*p *< 0.001, compared with “SNL + DMSO” group, n = 4) **b** ELISA analysis of the expression of spinal TNF-α, IL-1β, and IL-6 in SNL + DMSO, and SNL + PDTC group. ^($^ *p *< 0.05, ^$$^*p *< 0.01, ^$$$^ *p *< 0.001 compared with “SNL + DMSO” group, n = 4)

To further investigate the role of NF-κB pathway in SNL, we performed intrathecal administration of PDTC, a highly effective inhibitor of NF-κB, to block the activation of spinal NF-κB. PDTC, which was administrated in the early phase of SNL, blocked the activation of NF-κB and prevented the induction of spinal MMP-9. However, PDTC had no effect on spinal ANXA10 expression neither in the early nor in the late phase of SNL. Western blot showed that in the late phase of SNL, MMP-9 was slightly inhibited by PDTC administration, but the difference was not significant (Fig. 5a). Additionally, the increase of relevant proinflammatory cytokines (TNF-α, IL-1β, and IL-6) was prevented by PDTC, which was administrated both in the early and late phase of SNL (Fig. 5b).

## Discussion

The main findings of this study are: (1) SNL caused pain hypersensitivity in rats, along with increased expression of spinal ANXA10, pNF-κB, and particular cytokines (TNF-α, IL-1β, and IL-6) both in the early and late phase of NP. (2) Knockdown of spinal ANXA10 suppressed the SNL-induced hyperalgesia and blocked the activation of NF-κB, TNF-α and IL-1β both in the early and late phase of NP. Meanwhile, spinal MMP-9 expression was slightly increased in the early phase, which could be suppressed by knockdown of ANXA10. In addition, knockdown of spinal ANXA10 could only inhibit the upregulation of spinal IL-6 in the late phase of SNL-induced NP. (3) Inhibition of NF-κB prevented the activation of MMP-9 in the early phase, and blocked the expression of pNF-κB, TNF-α and IL-1β both in the early and late phase of NP.

Neuropathic pain is a refractory disease that causes serious financial burden. Current therapeutic strategies, such as opioids and non-steroidal anti-inflammatory drugs (NSAIDs), are limited for long-term application by their serious side effects [23]. Thus, exploring the entire and exact mechanisms of neuropathic pain to search for new and effective therapy is very urgent. The underlying mechanisms involved in NP are quite complicated, involving neuroinflammatory responses, neuroimmunology, the formation of neuroplasticity, which contributes to peripheral and central sensitization [2, 24, 25].

The expression of many different kinds of genes in the spinal cord was screened by microarray method after SNL. Among the significant up-regulated genes, ANXA10 increased by over 70 times, which was the only significantly up-regulated member of annexins family [6]. Nerve damage or tissue inflammation induces the expression of inflammatory factors in the spinal cord and dorsal root ganglia. ANXA10 is widely distributed in spinal astrocytes and neurons, which could express inflammatory factors. The activation of particular inflammatory cytokines could act on neuronal excitability and accelerate the development of neuropathic pain. The release of inflammatory factors can be regulated by intracellular signaling pathways such as mitogen-activated protein kinase (MAPK), protein kinase, and NF-κB [26, 27]. NF-κB is the upstream signal of MMP-9 and promotes the activation of some proinflammatory cytokines in the process of NP [28]. There is a very high similarity of the structure of the ANX protein family. The highly conserved C-terminal core region of each ANX protein generally has four Anx repeats and Ca^2+^—binding sites that function in conjunction with other molecules and structures [29]. ANXA2, also a member of the annexins family, formed a complex with the subunit and increased the transcriptional activity of NF-κB, leading to upregulation of the transcription of several target genes, including the proinflammatory factor IL-6 [30]. Given the similar structure of ANXA2 and ANXA10, and most importantly, the importance of spinal ANXA10 and NF-κB in regulating neuropathic pain, we hypothesized that activation of spinal NF-κB/MMP-9 pathway and the downstream cytokines (TNF-α, IL-1β, and IL-6) underlies the mechanism of ANXA10 in modulating neuropathic pain. One of the most important findings of our study is that SNL caused pain hypersensitivity and increased the activation of spinal ANXA10/NF-κB, and cytokines (TNF-α, IL-1β, and IL-6) both in the early and late phase of NP. Besides, our study indicates that MMP-9 activity in the spinal cord dorsal horn was relatively low, which was moderately increased by SNL. MMP-9 may act as the downstream target of ANXA10/NF-κB pathway in the development rather than the maintenance of NP.

It is noteworthy that in our study, the expression level of spinal ANXA10 mRNA upregluated for nearly 100 times in SNL rats, but the protein only increased for 2 times. There may be two main reasons accounting for the different upregulation level of ANXA10 mRNA and protein. Firstly, the mRNA abundance of a particular gene does not necessarily have a linear relationship with the corresponding protein expression. There are many levels of regulation of gene expression, the regulation of transcriptional level is only a link. Post-transcriptional regulation and translation and post-translational regulation also play a role in the final amount of protein [31]. Furthermore, factors such as mRNA degradation, protein degradation, and folding of the expression may also result in inconsistent mRNA abundance and protein expression level [32]. Secondly, the expression levels of many genes vary with time, with peaks and bottoms [33]. If we adjust the time to detect the protein expression, maybe the increase range of protein expression is consistent with the mRNA expression. This will be further investigated in our future research.

Consequently, ANXA10 expression and its downstream pathways would be potential targets in treating neuropathic pain. The current study yields new insight into the potential molecular mechanisms in the spinal cord in neuropathic pain and may provide a scientific basis for clinical neuropathic pain control.

## Conclusions

Using SNL model, this study illustrates the involvement of ANXA10/NF-κB/MMP-9 and proinflammatory cytokines (TNF-α, IL-1β, and IL-6) in mediating NP at the level of spinal cord. MMP-9 may act as the downstream target of ANXA10/NF-κB pathway in the development rather than the maintenance of NP.

## Data Availability

The datasets analyzed during the current study are available from the corresponding author on reasonable request.

## Abbreviations

NP: neuropathic pain
SNL: spinal nerve ligation
ANXA10: annexin A10
NF-κB: nuclear factor kappa B
TNF-α: tumour necrosis factor-α
IL-1β: interleukine-1β
IL-6: interleukine-6
siRNA: small interfering RNA
PWT: paw withdrawal threshold
PWL: paw withdrawal latency
